# Case Report: AQP4-IgG-positive neuromyelitis optica spectrum disorder with CSF anti- NMDAR1 IgG positivity and encephalitic features

**DOI:** 10.3389/fimmu.2026.1834813

**Published:** 2026-07-13

**Authors:** Mingming Li, Lixia Chen, Yanjun Liu, Tiejun Gan, Jia Guo

**Affiliations:** 1 Department of Neurology, Lanzhou University Second Hospital, Lanzhou, Gansu, China; 2 Department of Nuclear Magnetic Resonance, Lanzhou University Second Hospital, Lanzhou, Gansu, China

**Keywords:** anti-NMDAR encephalitis, area postrema syndrome, case report, inebilizumab, neuromyelitis optica spectrum disorder

## Abstract

**Background:**

Overlap between aquaporin-4 immunoglobulin G (AQP4-IgG)-positive neuromyelitis optica spectrum disorder (NMOSD) and anti-N-methyl-D-aspartate receptor (NMDAR) encephalitis is uncommon and may be difficult to recognize, especially when NMOSD initially presents with brain-predominant lesions and nonspecific symptoms.

**Case presentation:**

A 47-year-old woman initially presented with anorexia, dizziness, nausea, and intractable vomiting. Brain MRI showed a non-enhancing lesion along the anterior wall of the third ventricle involving the hypothalamic region and adjacent periependymal diencephalon, and she was initially treated for possible Wernicke encephalopathy with only partial transient improvement. Seven months later, she developed bilateral blurred vision, and repeat MRI showed persistent diencephalic involvement together with a new lesion in the left cerebellar hemisphere. Over the following three months, she developed progressive cognitive decline and neuropsychiatric symptoms. Neuro-ophthalmologic assessment supported bilateral optic neuritis. Brain MRI later revealed bilateral thalamic and mesial temporal abnormalities, while cervical MRI disclosed a subtle dorsal medullary lesion corresponding to the area postrema. Cerebrospinal fluid (CSF) showed no pleocytosis or protein elevation, but serum and CSF were positive for AQP4-IgG, and CSF anti-NMDAR1 IgG was positive at a low titer (1:10). The patient was treated with high-dose intravenous methylprednisolone followed by oral prednisone taper and maintenance inebilizumab. During follow-up, cognition, mood, and visual acuity improved substantially, and serial MRI showed no new lesions.

**Conclusion:**

This case highlights that persistent vomiting with a periependymal diencephalic or area postrema-region lesion may indicate brain-predominant AQP4-IgG-positive NMOSD rather than nutritional encephalopathy alone. In patients with AQP4-IgG-positive NMOSD who later develop prominent psychiatric or cognitive deterioration with mesial temporal-thalamic abnormalities, CSF anti-NMDAR1 IgG positivity with encephalitic features should be interpreted carefully as a possible overlap rather than definite anti-NMDAR encephalitis. Correlation of the clinical course, serial MRI findings, and CSF and serum antibody results may help support earlier diagnosis and treatment in autoimmune overlap syndromes.

## Introduction

Neuromyelitis optica spectrum disorder (NMOSD) is an autoimmune astrocytopathy associated with pathogenic AQP4-IgG. Although optic neuritis and longitudinally extensive myelitis are its classic manifestations, AQP4-IgG-positive NMOSD may also present with brain-predominant lesions involving periependymal diencephalic, brainstem, and cerebellar regions. Such presentations may delay diagnosis when early symptoms are nonspecific and opticospinal features are initially absent (1).

Anti-NMDAR encephalitis is a distinct antibody-mediated encephalitis characterized by psychiatric symptoms, cognitive dysfunction, seizures, movement disorders, and autonomic instability. Overlap between anti-NMDAR encephalitis and demyelinating syndromes has increasingly been recognized but overlap with AQP4-IgG-positive NMOSD remains uncommon. Distinguishing true overlap from incidental antibody positivity can be challenging, especially when antibody titers are low and the clinical phenotype is atypical.

Here, we describe a woman with brain-predominant AQP4-IgG-positive NMOSD who later developed neuropsychiatric and cognitive deterioration together with CSF anti-NMDAR1 IgG positivity, raising concern for a possible overlap. This case shows the value of relating lesion distribution to the clinical presentation and antibody findings in autoimmune overlap disorders, and it also supports early immunotherapy once the diagnosis is suspected.

## Case description

A 47-year-old Chinese woman presented in December 2023 with a 2-week history of anorexia, dizziness, nausea, and intractable vomiting. She was initially admitted to the gastroenterology department. Brain MRI revealed a lesion along the anterior wall of the third ventricle, and Wernicke encephalopathy was considered. Parenteral vitamin B1, B6, and B12 therapy produced only partial and transient improvement. Over the subsequent month, she developed depressive and anxiety symptoms, insomnia, occipital pressure-type headache, intermittent blurred vision, and a 5-kg weight loss. Her medical history included hypertension for 1 year and type 2 diabetes mellitus for 6 months. There was no relevant family history. Initial laboratory evaluation showed antinuclear antibody positivity, mildly elevated free thyroxine, positive thyroid-stimulating hormone receptor antibodies, hyperglycemia, and glucosuria.

Brain MRI performed on December 27, 2023, showed a T1-hypointense lesion with corresponding FLAIR hyperintensity along the anterior wall of the third ventricle, predominantly on the left, involving the periependymal diencephalon and the anterior commissure-hypothalamic region. No definite abnormal cerebellar signal was seen at the level of the posterior fossa at that time.

On July 31, 2024, she presented again with one week of bilateral blurred vision. Repeat MRI showed persistent hyperintensity along the anterior wall of the third ventricle together with a new hyperintense lesion in the left cerebellar hemisphere. Post-contrast imaging at the same level showed no definite enhancement of the left cerebellar lesion (**Figure 1**). Immunotherapy was not initiated at that time.

**Figure 1:**
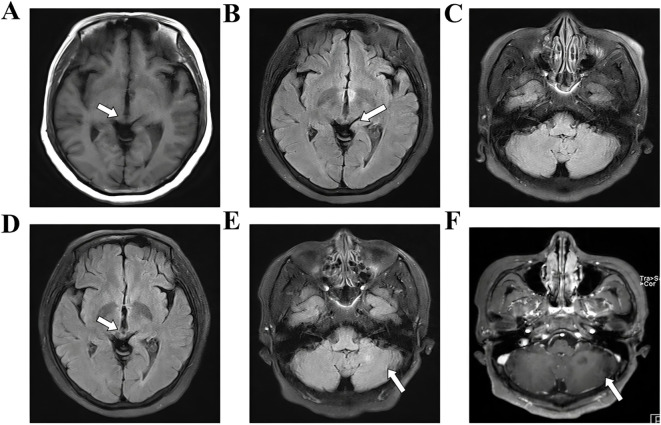
Annotated serial brain MRI showing initial diencephalic involvement and subsequent posterior fossa lesion development. **(A–C)** MRI obtained on December 27, 2023. **(A)** Axial T1-weighted image shows a hypointense lesion along the anterior wall of the third ventricle, indicated by the arrow. **(B)** Axial FLAIR image shows a left-predominant hyperintense lesion involving the periependymal diencephalon/anterior commissure-hypothalamic region, indicated by the arrow. **(C)** Axial FLAIR image at the level of the posterior fossa shows no definite abnormal signal in the cerebellum. **(D–F)** MRI obtained on July 31, 2024. **(D)** Axial FLAIR image shows persistent hyperintensity along the anterior wall of the third ventricle, indicated by the arrow. **(E)** Axial FLAIR image shows a new hyperintense lesion in the left cerebellar hemisphere, indicated by the arrow. **(F)** Axial contrast-enhanced T1-weighted image at the same level shows no definite enhancement of the left cerebellar lesion, with the lesion location indicated by the arrow.

During the following three months, she developed progressive cognitive decline and worsening neuropsychiatric symptoms, which led to readmission for further evaluation. Neuro-ophthalmologic assessment showed visual acuity of 0.07 in the right eye and 0.10 in the left eye. Optical coherence tomography demonstrated retinal nerve fiber layer thicknesses of 91 μm and 97 μm, and visual evoked potentials showed bilateral P100 latency prolongation with preserved amplitudes. Ophthalmologic evaluation supported bilateral optic neuritis. Cervical MRI disclosed a subtle dorsal medullary hyperintensity corresponding to the area postrema, whereas thoracic spine MRI showed no definite intramedullary lesion. CSF analysis showed no pleocytosis or protein elevation, with an opening pressure of 145 mmH_2_O and protein of 0.19 g/L. Microbiological studies, including cultures and India ink staining, were negative. CSF-restricted oligoclonal bands were present, indicating intrathecal IgG synthesis. Cognitive screening showed marked impairment, with an MMSE score of 15/30 and a MoCA score of 14/30. Brain MRI revealed bilateral thalamic hyperintensities, persistent posterior fossa involvement including the left cerebellar hemisphere and adjacent middle cerebellar peduncle, and mild irregular enhancement in mesial temporal lesions (**Figure 2**). Serum AQP4-IgG was positive at 1:32, and CSF AQP4-IgG was positive at 1:3.2 by live cell-based assay. Serum MOG-IgG was negative by cell-based assay. CSF anti-NMDAR1 IgG, targeting the NR1 subunit of the NMDAR, was positive at a low titer of 1:10 using a transfected cell-based assay, whereas serum anti-NMDAR1 IgG was negative at the diagnostic reassessment. Systemic tumor screening, including pelvic ultrasound and abdominopelvic CT, showed no evidence of ovarian teratoma or other malignancy. The combination of persistent nausea and vomiting, an area postrema-region lesion, periependymal diencephalic involvement, cerebellar and middle cerebellar peduncle lesions, objective bilateral optic pathway involvement, and concordant serum and CSF AQP4-IgG positivity was consistent with AQP4-IgG-positive NMOSD. The diagnosis was anchored to the established 2015 International Panel for NMO Diagnosis criteria and was further supported by the revised IPND 2025 framework (1, 2). The later appearance of prominent psychiatric and cognitive symptoms, together with mesial temporal and thalamic abnormalities on MRI and CSF anti-NMDAR1 IgG positivity, raised concern for a coexisting encephalitic process. However, given the low CSF anti-NMDAR1 IgG titer, initially negative serum anti-NMDAR1 IgG, absence of seizures, movement disorders, or autonomic instability, and lack of CSF pleocytosis or protein elevation, the anti-NMDAR-related component was interpreted cautiously. Therefore, the case was considered AQP4-IgG-positive NMOSD with CSF anti-NMDAR1 IgG positivity and encephalitic features, representing a possible overlap rather than definite anti-NMDAR encephalitis overlap.

**Figure 2:**
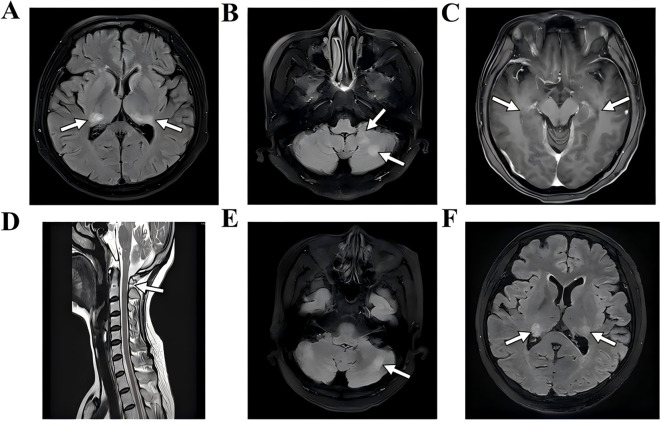
Annotated MRI during hospitalization and follow-up. **(A–D)** MRI obtained on November 10, 2024. **(A)** Axial FLAIR image shows bilateral thalamic hyperintensities, indicated by arrows. **(B)** Axial FLAIR image shows hyperintensity involving the left cerebellar hemisphere and adjacent middle cerebellar peduncle, indicated by arrows. **(C)** Axial contrast-enhanced T1-weighted image shows mild irregular enhancement in the mesial temporal lesions, indicated by arrows. **(D)** Sagittal cervical MRI shows a subtle dorsal medullary hyperintensity corresponding to the area postrema region, indicated by the arrow. **(E, F)** Follow-up MRI obtained on November 18, 2025. **(E)** Axial FLAIR image shows stable nonprogressive abnormality in the left cerebellar hemisphere, indicated by the arrow. **(F)** Axial FLAIR image shows persistent but nonprogressive bilateral thalamic abnormalities, indicated by arrows, without new lesions.

The patient received intravenous methylprednisolone 1,000 mg daily with dose tapering every three days, followed by oral prednisone acetate 60 mg daily with gradual tapering. Given the brain-predominant AQP4-IgG-positive phenotype, relapse risk, and coexisting antibody-mediated encephalitic features, maintenance inebilizumab was initiated, with loading doses on days 1 and 15 followed by re-dosing every six months. At admission in November 2024, serum AQP4-IgG was positive at 1:32; it became negative in January 2025 and remained negative in February 2025. CSF anti-NMDAR1 IgG was positive at a low titer of 1:10 using a transfected cell-based assay, and subsequent serum testing in January 2025 detected low-titer anti-NMDAR1 IgG positivity at 1:1. Follow-up MRI obtained on November 18, 2025, showed stable nonprogressive abnormalities in the left cerebellar hemisphere and bilateral thalami, without new lesions. OCT/RNFL and VEP remained stable. Visual acuity improved to approximately 0.30-0.40. Cognitive function improved markedly, with MMSE increasing from 15 to 30 and MoCA from 14 to 25, and mood symptoms also improved substantially. There was no clinical relapse or radiologic progression during follow-up, and maintenance inebilizumab was continued with regular clinical, imaging, and serologic monitoring. The timeline of the clinical course is summarized in **Table 1**, and serial autoantibody testing results are summarized in **Table 2**.

**Table 1:** Timeline of the clinical course.

Date	Clinical events	Key investigations	Interpretation/management
Dec 2023	Anorexia, dizziness, nausea, intractable vomiting	Brain MRI on December 27, 2023: anterior third-ventricle and periependymal diencephalic lesion; no definite cerebellar abnormality at the posterior fossa level	Initially treated as possible Wernicke encephalopathy; partial transient response to B vitamins
Jan 2024	Depression, anxiety, insomnia, headache, intermittent blurred vision, weight loss	Autoimmune and endocrine screening	Course became atypical for isolated nutritional encephalopathy
Jul 31, 2024	Bilateral blurred vision	Brain MRI on July 31, 2024: persistent diencephalic lesion and new left cerebellar lesion	Brain-predominant inflammatory demyelinating process considered
Oct–Nov 2024	Progressive cognitive decline and neuropsychiatric worsening	MMSE 15/30, MoCA 14/30; ophthalmologic and VEP evidence of bilateral optic neuritis	Concern for NMOSD with additional encephalitic component
Nov 10, 2024	Hospital admission and diagnostic reassessment	Brain MRI: bilateral thalamic lesions, posterior fossa involvement, mesial temporal enhancement; cervical MRI: area postrema lesion; serum/CSF AQP4-IgG positive; CSF anti-NMDAR1 IgG 1:10	Working diagnosis: AQP4-IgG-positive NMOSD with CSF anti-NMDAR1 IgG positivity and encephalitic features; possible overlap considered
Nov 2024 onward	Acute immunotherapy and maintenance treatment	IV methylprednisolone, oral prednisone taper, inebilizumab	Clinical stabilization
Jan-Feb 2025	Early serologic follow-up	Serum AQP4-IgG became negative in January 2025 and remained negative in February 2025; low-titer serum anti-NMDAR1 IgG was detected in January 2025.	Continued maintenance therapy and surveillance
Nov 18, 2025	Imaging and clinical follow-up	Follow-up MRI: stable left cerebellar and bilateral thalamic abnormalities without new lesions; stable OCT/VEP; improved cognition and vision	No clinical or radiologic progression

**Table 2:** Serial autoantibody testing results.

Time point	Specimen	AQP4-IgG	Anti-NMDAR1 IgG
Jan 2024	Serum	Not tested	Not tested
Nov 2024 diagnostic reassessment	Serum	Positive, 1:32	Negative
Nov 2024 diagnostic reassessment	CSF	Positive, 1:3.2	Positive, 1:10
Jan 2025 follow-up	Serum	Negative	Positive, 1:1
Feb 2025 follow-up	Serum	Negative	Not tested

## Discussion

AQP4-IgG-positive NMOSD may present with brain-predominant lesions involving periependymal, brainstem, and cerebellar regions, and anatomically concordant syndromes should prompt consideration of AQP4-related disease even when opticospinal manifestations are not dominant (1). In this patient, recurrent nausea and vomiting, a periependymal diencephalic lesion along the anterior wall of the third ventricle, later cerebellar and middle cerebellar peduncle involvement, an area postrema-region lesion, and objective optic pathway dysfunction were all in keeping with a brain-predominant AQP4-IgG-positive NMOSD phenotype. The diagnosis was primarily anchored to the established 2015 IPND criteria. The recently proposed IPND 2025 framework also supports classifying this presentation within AQP4-IgG-positive NMOSD, particularly given the lesion distribution, visual pathway involvement, and antibody findings (2). Wernicke encephalopathy was initially considered because of persistent vomiting and partial transient response to parenteral vitamins; however, the absence of classic mammillary body or periaqueductal involvement, the subsequent lesion evolution, and the incomplete clinical response argued against isolated nutritional encephalopathy (3).

Anti-NMDAR encephalitis often presents with psychiatric symptoms, behavioral change, and cognitive impairment, whereas MRI may be normal or show relatively mild abnormalities on T2-weighted and FLAIR images involving the mesial temporal lobes, thalami, or other limbic-associated regions (4). In our patient, progressive neuropsychiatric and cognitive deterioration together with bilateral thalamic and mesial temporal abnormalities on MRI suggested an encephalitic process beyond NMOSD alone. CSF anti-NMDAR1 IgG was positive at a low titer, whereas serum testing was initially negative. Although a low antibody titer should be interpreted with caution, the temporal evolution of psychiatric and cognitive symptoms and the mesial temporal-thalamic MRI abnormalities suggested that CSF anti-NMDAR1 IgG positivity may have clinical relevance. Nevertheless, the evidence was insufficient to define a definite anti-NMDAR encephalitis overlap, and the case was therefore interpreted as AQP4-IgG-positive NMOSD with CSF anti-NMDAR1 IgG positivity and encephalitic features. This interpretation is also supported by the published literature showing that anti-NMDAR encephalitis may overlap with demyelinating syndromes and that clinical context is essential when assigning significance to coexisting neural antibodies (5).

This case illustrates why antibody results should be interpreted together with the clinical course and MRI findings in autoimmune neuroinflammatory disease. Serum and CSF AQP4-IgG positivity explained the area postrema syndrome, periependymal diencephalic lesion, optic pathway involvement, and cerebellar-peduncular abnormalities, whereas the later emergence of marked psychiatric and cognitive dysfunction with mesial temporal-thalamic MRI changes was more consistent with an additional anti-NMDAR encephalitic component. In this setting, dual antibody positivity was not interpreted in isolation, but in conjunction with serial symptom evolution and anatomically concordant MRI findings. This is especially relevant when the initial presentation is nonspecific and may resemble a metabolic, nutritional, or endocrine disorder.

The therapeutic approach was guided by the likely humoral pathogenesis of both disease components (6). High-dose corticosteroids were used for the acute attack, followed by maintenance CD19-directed B-cell depletion with inebilizumab (7). This strategy was selected because inebilizumab is an established therapy for AQP4-IgG-positive NMOSD and offers broader depletion of CD19-lineage B cells and plasmablast-related compartments relevant to antibody-mediated disease (8). In addition, the ongoing phase 2B, randomized, double-blind ExTINGUISH trial provides an external prospective rationale for evaluating inebilizumab in anti-NMDAR encephalitis, although efficacy results are not yet available and the study should not be interpreted as proof of benefit at present (9). In our patient, neurological and visual recovery, together with stable follow-up MRI on November 18, 2025, was in line with a favorable treatment response.

This report has several limitations. First, the anti-NMDAR1 antibody titer was low, and not all classical features of anti-NMDAR encephalitis were present. Second, this was a single-case observation, so the contribution of each treatment component cannot be separated. Third, although the patient improved after immunotherapy, causality cannot be definitively established. Nevertheless, the chronological evolution, lesion topography, paired serum and CSF antibody findings, and sustained stabilization after treatment together support the diagnosis of AQP4-IgG-positive NMOSD with CSF anti-NMDAR1 IgG positivity and encephalitic features, representing a possible overlap rather than definite anti-NMDAR encephalitis overlap.

## Patient perspective

At the onset of the illness, the patient experienced persistent nausea and vomiting, sleep disturbance, blurred vision, and later memory and mood changes, which caused distress and interfered with daily life. After immunotherapy, she reported gradual improvement in vision, memory, mood, and daily functioning. The stable condition during follow-up gave her more confidence in the treatment plan.

## Conclusion

This case has several practical implications. Persistent vomiting with a periependymal diencephalic lesion or an area postrema-region lesion should prompt early AQP4-IgG testing, even when opticospinal features are not evident at onset. In patients with AQP4-IgG-positive NMOSD who later develop marked psychiatric or cognitive decline together with mesial temporal-thalamic abnormalities, CSF anti-NMDAR1 IgG positivity with encephalitic features should be considered and interpreted cautiously in the full clinical context. Careful correlation of symptom progression, MRI changes, and antibody results may help shorten diagnostic delay and support earlier treatment.

## Data Availability

The datasets presented in this study can be found in online repositories. The names of the repository/repositories and accession number(s) can be found in the article/supplementary material.
